# Potential supplementary tumor markers for liquid biopsy in non-small cell lung cancer

**DOI:** 10.3389/ebm.2025.10523

**Published:** 2025-05-29

**Authors:** Jin Xiang, Junyan Peng, Zhifang Xing, Guoqiang Ren, Huating Zhang, Xiaodong Song, Bo Zhang, Ming Guan, Guojun Cao

**Affiliations:** ^1^ Department of Laboratory Medicine, Huashan Hospital, Shanghai Medical College, Fudan University, Shanghai, China; ^2^ Tendering Office, Huashan Hospital, Shanghai Medical College, Fudan University, Shanghai, China; ^3^ Department of Pathology, Huashan Hospital, Shanghai Medical College, Fudan University, Shanghai, China

**Keywords:** digital PCR, liquid biopsy, tumor markers, EGFR mutation, non-small cell lung cancer

## Abstract

The identification of epidermal growth factor receptor (EGFR) tyrosine kinase (TK) domain mutations in non-small cell lung cancer (NSCLC) patients is crucial for therapeutic decision-making and monitoring EGFR-tyrosine kinase inhibitor (TKI) resistance. Liquid biopsy has emerged as a promising alternative for patients ineligible for invasive tissue sampling. This study investigated the clinical utility of a novel chip-based digital PCR (dPCR) platform for detecting two important EGFR mutations - exon 19 deletions (19del) and threonine-methionine amino acid substitution at position 790 (T790M) - in serum samples, while exploring potential serum biomarkers for mutation prediction. The collection of 350 serum samples were conducted on patients diagnosed with NSCLC at Huashan Hospital between August 2023 and February 2024. Cell-free deoxyribonucleic acid (cfDNA) was extracted from serum and was analyzed for EGFR mutations using dPCR. The serum tumor marker levels were quantified. The dPCR assay demonstrated positive predictive values of 73.33% for 19del and 28.57% for T790M. Biomarker analysis revealed a carbohydrate antigen (CA) 199 cutoff of 11.75 U/mL (AUC = 0.707, 95% CI: 0.573–0.841, *P* = 0.005) for 19del detection, while progastrin-releasing peptide (ProGRP) showed a cutoff of 45.15 pg/mL (AUC = 0.628, 95% CI: 0.521–0.735, *P* = 0.028) for T790M identification. Variant rate exhibited significant positive correlations with biomarker concentrations: 19del variant rates significantly associated with CA125 levels (r = 0.624, *P* = 0.010), while T790M correlated with both carcinoembryonic antigen (CEA) (r = 0.531, *P* = 0.004) and ProGRP (r = 0.395, *P* = 0.041) in mutation-positive cohorts. These findings indicate that serum-based dPCR liquid biopsy demonstrates potential clinical utility as a supplementary approach to tissue biopsy for NSCLC genotyping. Notably, elevated serum tumor marker levels correlate with enhanced mutation detection rates in liquid biopsy, implying their potential supplementary value in prioritizing patients for molecular profiling.

## Graphical Abstract

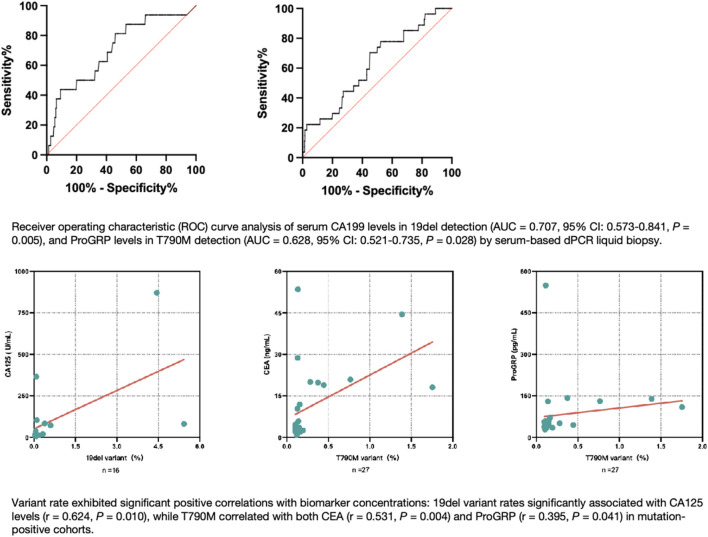

## Impact statement

The dPCR liquid biopsy technique is bringing a ray of hope for NSCLCs who are unable to provide sufficient tissue samples for genotyping. However, its widespread clinical adoption faces persistent challenges. Our study verified the analytical performance of a new chip-based dPCR technique platform for quantifying two critical EGFR mutations (19del and T790M) in serum-derived cfDNA, and concurrently identified the potential serum biomarkers to optimize patient stratification for cost-effective molecular profiling. Key findings revealed that elevated serum CA199 and ProGRP levels demonstrated predictive utility for 19del and T790M mutations, respectively. Positive correlations were observed between the 19del variant rate and CA125 level, as well as between the T790M variant rate and CEA, ProGRP level.

## Introduction

Lung cancer is the most common cancer type and leading cause of cancer-related deaths in China and worldwide [1]. NSCLC constitutes approximately 75%–80% of all lung cancer cases [2]. Because it is often asymptomatic in its early phases, most patients are diagnosed with NSCLC at an advanced stage with no possible surgical intervention, resulting in poor prognosis [3]. In oncogene-driven NSCLC, accurate molecular subtyping is the prerequisite for precision treatment. Mutations in the EGFR gene are one of the most common oncogenic driver mutations in non-squamous NSCLC, with a positive rate of 50% in East Asian populations [4]. For NSCLC tumors harboring EGFR mutations, especially in advanced patients, EGFR-TKI-based therapy has become the standard treatment approach. This has been shown to be superior to chemotherapy for improving patient survival and prognosis [5]. As one of the most important and common EGFR mutations, 19del constitutes approximately 85% of all EGFR mutations together with exon 21 L858R point mutations. These alterations, often referred to as “classic” mutations, predicts good response to EGFR-TKIs [6]. However, more than 50% of patients receiving the first- or second-generation TKIs develop a point mutation in EGFR that results in T790M 9–14 months after treatment, leading to drug resistance [7]. Therefore, new therapeutic methods are needed for these NSCLC patients. Currently, EGFR gene mutation testing requires tumor tissue acquired by surgery or biopsy. However, the clinical application of tumor tissue biopsy is limited. Sampling is highly invasive and cannot be used for the dynamic monitoring of tumors during treatment and follow-up. Moreover, tumor tissue heterogeneity may lead to certain mutant genes not being detected in some patients. Liquid biopsy is expected to bring new hope as a non-invasive and highly sensitive approach for NSCLC treatment guidance and monitoring. This minimally invasive and rapid technique enables real-time decision-making in various clinical scenarios by isolating, detecting, and analyzing tumor-released nucleic acids circulating in body fluids [3, 8, 9]. Peripheral blood samples can properly represent the tumor origin and are easy to obtain, rendering them as optimal materials for liquid biopsy. Circulating cfDNA in the peripheral blood of tumor origin, called circulating tumor DNA (ctDNA), is tumor-specific and can be representative of the full tumor tissue composition. However, ctDNA accounts for less than 1% of cfDNA and is highly fragmented with a short half-life. The proportion of ctDNA decreases significantly in early tumors and after treatment responses [10]. Therefore, a highly sensitive detection method is crucial for clinical practice.

Technologies based on polymerase chain reaction (PCR), including super amplification refractory mutation system (super-ARMS), beads, emulsion, amplification, magnetics (BEAMing), and dPCR, are suitable for detecting known specific loci and can all be used to detect cfDNA. As the latest generation of PCR, dPCR assay builds upon traditional PCR amplification and fluorescent probe-based detection methods is able to provide precise and absolute quantification of cfDNA mutations with good analytical sensitivity [11]. The target concentration was calculated according to Poisson distribution by directly counting the partitions that contained fluorescent target molecules. Several studies have identified the utility of liquid biopsy in the identification of EGFR mutations and acquired resistance with good sensitivities for various blood-based biomarkers. However, the clinical application and promotion of liquid biopsy are faced with similar difficulties at present, which including differences in methodological sensitivity and specificity of the assay itself, economic factors and the difficulties in conducting experiments in some laboratories. Recent studies have focused on developing predictive models that leverage clinical, radiological, and laboratory characteristics to ascertain EGFR mutation status in NSCLC [12]. This study aimed to use a new chip-based dPCR technique platform for the quantification of circulating cfDNA targets (EGFR 19del and T790M) in NSCLC patient serum samples, and to find potential serum biomarkers which may help to screen beneficiary patients and improve the dPCR liquid biopsy detection efficiency.

## Materials and methods

### Patients and materials

In this study, serum samples were obtained from 350 patients diagnosed with NSCLC. All samples were sent to the Department of Laboratory Medicine of Huashan Hospital for immunological testing between August 2023 and February 2024. The patients’ demographic and clinical data were retrospectively investigated from an electronic medical records system. The data included sex, age, pathological classification, disease stage, tissue gene status, and the process of EGFR-TKI treatment. At least 4.0 mL of venous blood from each patient was collected in BD Vacutainer ^®^ SST™ blood collection tubes (Becton, Dickinson and Company, USA) with gel separators and silica coagulant. Sampling and centrifugation were performed according to standard operating procedures. Serum samples were included if they met the following criteria: obtained from a donor who was histopathologically diagnosed with NSCLC, had no obvious hemolysis and lipemia, and was no less than 1.0 mL in volume. The selected samples were collected and stored in 1.5 mL Eppendorf (EP) tubes at −80°C until use. All procedures were in accordance with the Helsinki Declaration. The protocol of the current study was reviewed and approved by Huashan Hospital Ethical Committee (2022-572) and informed written consent was obtained from all enrolled patients.

### cfDNA extraction

Purified nucleic acids were extracted from 1.0 mL of thawed and higher-speed centrifuged serum using a High Pure Viral Nucleic Acid Large Volume Kit (column method, Cat. No. 05114403001, Roche Diagnostics, Mannheim, Germany) according to the manufacturer’s instructions with an elution volume of 70 μL.

### EGFR mutation detection with the dPCR assay

Under standard conditions, dPCR was performed using a Digital LightCycler^®^ System (Roche Diagnostics GmbH) to separately amplify 19del and T790M of EGFR. A maximum of 37 μL serum cfDNA, 10 μL Digital LightCycler^®^ 5× DNA Master Mix, 0.5 μL restriction enzyme (HaeIII), 2.5 μL Parameter-Specific Reagents (PSR, containing premixed primers and probes), and PCR-grade water were added to the reaction mixture to a total volume of 50 μL. As recommended to enable a higher sensitivity for detecting the target sequences, the high sensitivity nanowell plate with approximately 20,000 partitions per reaction was used for a higher input volume per lane. With this nanowell plate, 45 μL of reaction mixture were added to each lane before loading and partitioning the plate.

The 19del mutation site was analyzed using two labeled probes: 1) a reference probe (HEX-labeled), which was designed to bind to the amplicon irrespective of mutation presence, and 2) an indel probe (FAM-labeled), which was designed to bind to the wild-type (WT) sequence but not to any of the mutated sequences. The assay allows the detection of 28 deletions in the EGFR exon 19 (with COSMIC IDs: COSM26038, COSM13550, COSM6223, COSM13552, COSM13551, COSM12385, COSM6225, COSM12728, COSM12678, COSM12386, COSM12416, COSM12367, COSM12384, COSM18427, COSM12422, COSM12419, COSM23571, COSM6220, COSM6218, COSM12382, COSM12383, COSM6254, COSM12403, COSM6255, COSM12387, COSM6210, COSM12369, COSM12370). The 19del-PCR reaction was performed using the following cycling conditions: stage 1: 50°C for 2 min; stage 2: 95°C for 2 min; stage 3: 40 cycles of 95°C for 15 s and 60°C for 30 s; stage 4: 40°C for 30 s. The T790M mutation site (COSMIC ID: COSM6240) was analyzed using two labeled probes binding competitively to the mutation site. The mutant probe is FAM-labeled and the WT probe is HEX-labeled. The T790M-PCR reaction was performed using the following cycling conditions: stage 1: 50°C for 2 min; stage 2: 95°C for 2 min; stage 3: 40 cycles of 95°C for 10 s and 58°C for 20 s; stage 4: 40°C for 30 s. Finally, the Digital LightCycler^®^ Development Software was used to perform partition clustering and analyze the results. The 19del mutation results were analyzed using two-dimensional (2D) scatter plots and the T790M mutation results were analyzed using one-dimensional (1D) scatter plots. A sample was considered mutant-positive if its gene variant rate was above the corresponding cutoff (0.05% for 19del mutation and 0.10% for T790M mutation) as provided by the manufacturers.

### Analytical performance verification of the dPCR assay

The sensitivity of the EGFR 19del and T790M dPCR systems were evaluated using simulation samples with gradient mutation loads (5%, 1%, 0.5%, 0.1%, 0.05%), which were generated by mixing a mutant plasmid and the WT plasmid that provided by the manufacturer at different proportions. Three replicates per batch were used for each mutation load. Samples with only genomic DNA (0% mutant) were also included as a control. Positive 19del simulation samples, positive T790M simulation samples, and genomic DNA samples from the peripheral blood of healthy individuals were used to test the specificity of the EGFR 19del and T790M dPCR systems.

### Tumor marker quantification

The concentrations of tumor markers associated with lung cancer, including CEA, cytokeratin 19 fragment (Cy211), neuron specific enolase (NSE), ProGRP, squamous cell carcinoma antigen (SCCA), CA199, CA125, CA153, CA724, and alpha1-fetoprotein (AFP), in residual serum samples were analyzed by electrochemiluminescence immunoassays (ECLIAs) on a cobas^®^ 8000 modular analyzer series e801 (Roche Diagnostics GmbH) following the manufacturer’s instructions. The reference intervals of these 10 serological markers were <6.5 ng/mL, <3.3 ng/mL, <17 ng/mL, <69.2 pg/mL, <2.7 ng/mL, <37 U/mL, <35 U/mL, <25 U/mL, <8.2 U/mL, and <7 ng/mL, respectively.

### Statistical analysis

Statistical analyses were performed using IBM SPSS Statistics Version 25.0 (IBM Corp., Armonk, NY, USA) and Microsoft Excel Version 2013 (Microsoft Corp., Redmond, WA, USA). The figures were generated using GraphPad Prism 9.2 (GraphPad Software Inc., La Jolla, CA, USA).

Data for continuous variables are presented as the median with interquartile range (IQR), while data for categorical variables are presented as numbers and percentages. Comparisons between groups were performed by applying the chi-squared test, McNemar test, or Mann-Whitney U test, with correlations being analyzed by applying the Spearman test. A two-sided *P*-value <0.05 was considered statistically significant. ROC curve analysis and the Youden index were used to determine the best cutoff value and obtain the corresponding sensitivity and specificity values.

## Results

### General characteristics of patients

In this study, the frequencies of EGFR 19del and T790M mutations in serum cfDNA from 350 NSCLC patients were determined. The clinical characteristics of the study participants are shown in Table 1. Among them, 217 patients were male and 133 were female. The median age was 63 (56–70) years, with 300 patients diagnosed with adenocarcinoma, 34 with squamous carcinoma, and 16 with other subtypes (including large cell carcinoma, sarcomatoid carcinoma, and poorly-differentiated NSCLC). The numbers of cases with stage I, II, III, and IV disease were 18, 12, 26, and 197, respectively, with 97 cases not yet staged. Tissue molecular tests were performed on samples from 56.0% (196 of 350) of the patients. The re-biopsy rate was 19% (38 of 196). The median time interval between tissue biopsy and blood sampling was 16.5 (6–32) months. For NSCLC treatment, 127 patients underwent EGFR-TKI therapy for 17 (8–33) months. Among these cases, 48 patients started with a first- or second-generation EGFR-TKI, with 28 of them switching to a third-generation EGFR-TKI during later treatment. Additionally, 79 cases started their treatment with the third-generation EGFR-TKIs (instead of starting with first- or second-generation and then switching), 14 of whom were treated with a combination of more than one EGFR-TKI.

**TABLE 1:** Clinical characteristics of the EGFR mutant-positive and EGFR mutant-negative participants.

Features	dPCR- 19del		*p* value	dPCR- T790M		*p* value
	Negative (n = 331)	Positive (n = 19)		Negative (n = 322)	Positive (n = 28)	
Sex			0.71			0.80
Male, n	206	11		199	18	
Female, n	125	8		123	10	
Age, year, median (IQR)	63 (57–70)	63 (48–76)	0.92	64 (56–70)	61 (58–68)	0.61
Pathology			0.63			0.096
Adenocarcinoma, n	283	17		279	21	
Others, n	48	2		43	7	
Stage			**0.041**			0.34
I, n	18	0		18	0	
II, n	11	1		12	0	
III, n	21	5		23	3	
IV, n	186	11		178	19	
N/A, n	95	2		91	6	
EGFR-TKI therapy			**<0.001**			0.51
“First or second” generation TKIs, n	46	2		44	4	
“Third generation” TKIs, n	67	12		75	4	
Never, n	211	5		196	20	
N/A, n	7	0		7	0	

EGFR, epidermal growth factor receptor; dPCR, digital polymerase chain reaction; IQR, interquartile range; TKI, tyrosine kinase inhibitor; N/A, not available; first-generation TKIs, gefitinib, icotinib, erlotinib; second-generation TKIs, afatinib, dacomitinib; third-generation TKIs: osimertinib, almonertinib, vormetinib. Bold values indicate statistical significance (*p* < 0.05).

### Performance of the dPCR assay in detecting EGFR 19del and T790M mutations

**FIGURE 1:**
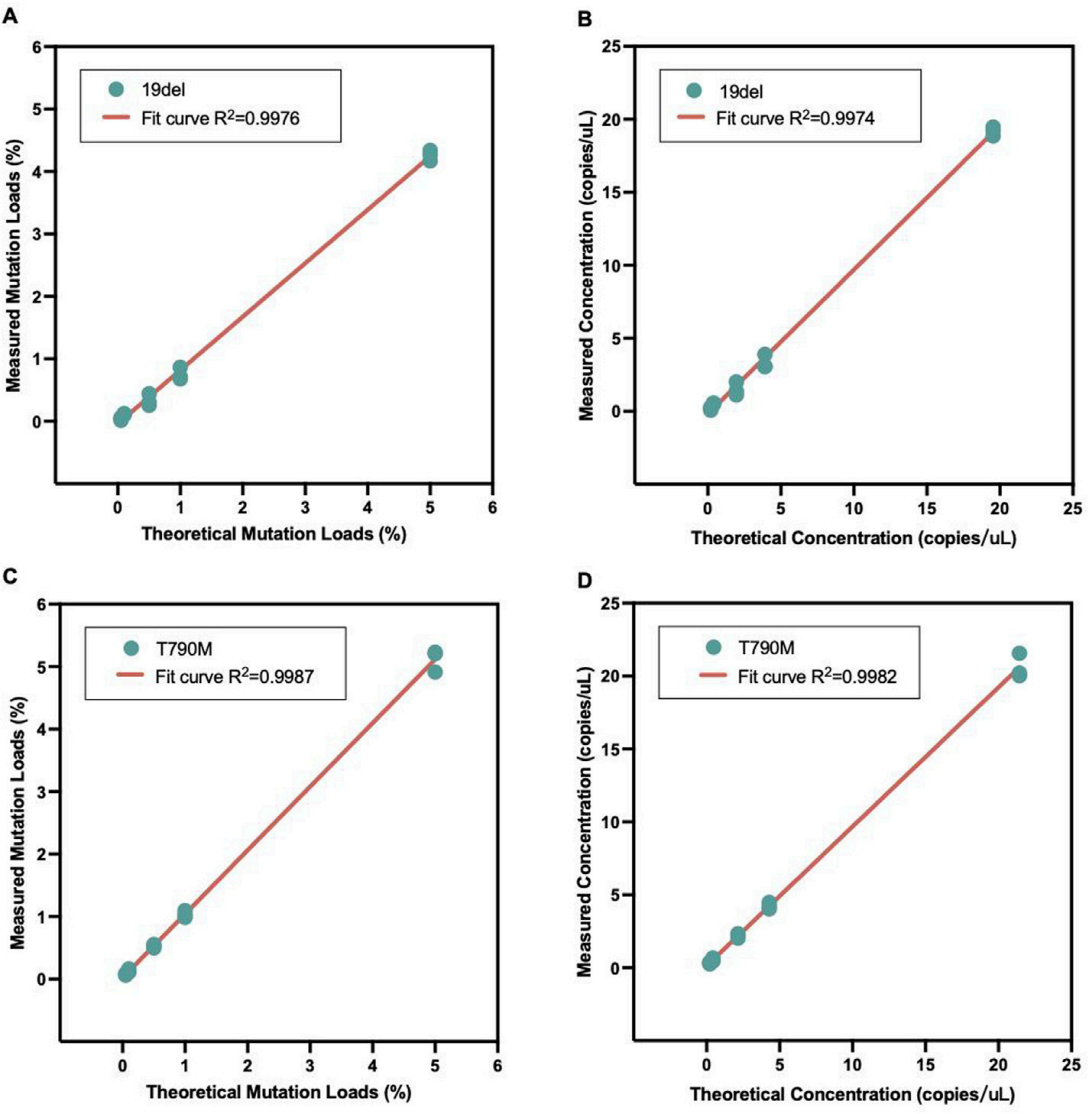
Sensitivity values of the digital polymerase chain reaction (dPCR) platform. The solid line represents the best fit line for mutation load data. The goodness of fit value (R squared) is shown in each figure panel. **(A)** Mutation load results for the EGFR 19del mutation. **(B)** Quantitative concentration results for 19del. **(C)** Mutation load results for the EGFR T790M mutation. **(D)** Quantitative concentration results for T790M.

For the mutational analysis, firstly the quantitative performance of the dPCR assay was evaluated. The sensitivity values of different targets were evaluated by mutation load. The results suggested that the mutant allele detection was quantitative and linear, with the limit of detection for EGFR 19del and T790M mutations being 0.05% and 0.10% mutation load, respectively (Figure 1). The dPCR system amplified the target nucleic acid template specifically, as no non-specific amplification of the background DNA or other DNA with similar sequences was observed.

**FIGURE 2:**
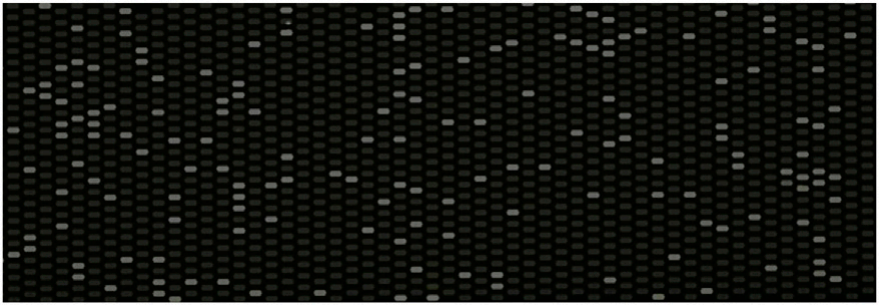
Image of the digital polymerase chain reaction (dPCR) nanowell plate after partitioning and amplification. Target-positive partitions are visible as discrete fluorescent wells against a background of non-fluorescent (target-negative) wells.

**FIGURE 3:**
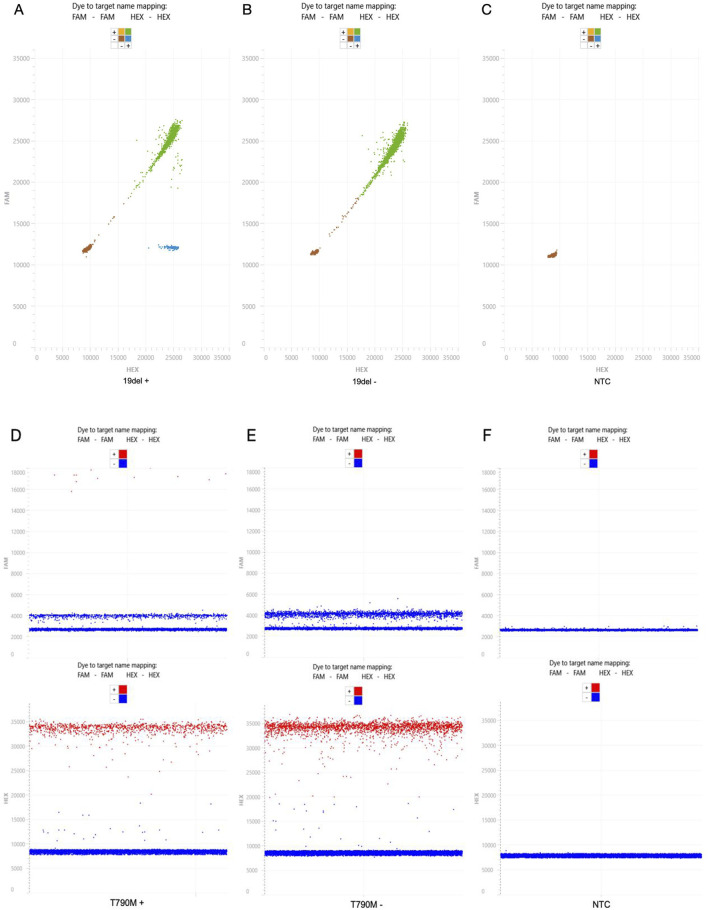
Two-dimensional (2D) and one-dimensional (1D) scatter plots of the positive, negative, and no template control (NTC) results of the EGFR 19del and T790M mutations using the digital polymerase chain reaction (dPCR) assay. The NTC samples were composed of only PCR-grade water. **(A)** 2D scatter plot of an exon 19del-positive sample, with a variant rate of 4.4467%, 2.9903 variant copies/μL, and 64.2565 wild-type (WT) copies/μL. **(B)** 2D scatter plot of an exon 19del-negative sample, with a variant rate of 0%, 0 variant copies/μL, and 139.3881 WT copies/μL. **(C)** 2D scatter plot of the 19del NTC, with a variant rate of 0%, 0 variant copies/μL, and 0 WT copies/μL. **(D)** 1D scatter plots of a T790M-positive sample, with a variant rate of 1.39%, 1.0052 variant copies/μL, and 71.4508 WT copies/μL. **(E)** 1D scatter plots of a T790M-negative sample, with a variant rate of 0%, 0 variant copies/μL, and 97.4854 WT copies/μL. **(F)** 1D scatter plots of the T790M NTC, with a variant rate of 0%, 0 variant copies/μL, and 0 WT copies/μL.

Next, the complete workflow was analyzed. EGFR 19del mutations were identified in 5.4% of the analyzed serum cfDNA samples (19 positives among 350 tested serum samples), while EGFR T790M mutations were observed in 8.0% of the cases (28 of 350). The maximum variant rate was 5.4239% for 19del and 1.7539% for T790M. Figure 2 shows an image of the dPCR nanowell plate after the partitioning and amplification procedures. The plate was processed using the Digital LightCycler^®^ analysis algorithm. Furthermore, Figure 3 shows the 2D and 1D scatter plots for partition fluorescence and demonstrates the detection of each mutation in its corresponding channel. In the 2D scatter plots, the red partitions were double negative for HEX and FAM, the green partitions were double positive for HEX and FAM, the blue partitions were positive for only HEX, and the yellow partitions were positive for only FAM but none were observed. In the 1D scatter plots, the red partitions were positive and blue partitions were negative for that channel. The dPCR assay also enabled quantitative detection of gene mutations. The median concentrations of the mutant and WT copies of the 19del-positive samples were 0.07 (0.03–0.56) copies/μL and 64.26 (42.17–147.65) copies/μL, respectively. These showed significant differences when compared with the 19del WT samples (*P* < 0.001*, P* = 0.013). For T790M, the median concentrations of variant and WT copies were 0.1225 (0.0805–0.1583) copies/μL and 87.8397 (67.6016–110.0498) copies/μL, respectively. Only the former was statistically significantly higher when compared with the T790M WT samples (*P* < 0.001, *P* = 0.193).

Table 1 also shows the clinical characteristics of the patients after they were categorized by their positive or negative status for the EGFR 19del and T790M mutations. Among the 19del mutant-positive patients, 58% (11 of 19) were male and 90% (17 of 19) were diagnosed with adenocarcinoma. The median age was 63 (48–76) years. A statistically significant effect of different disease stages on serum 19del mutation detection was observed (*P* = 0.041). The positive rate of serum 19del detection was highest in patients who used the third-generation EGFR-TKIs compared with those who never used them or received other generations of EGFR-TKI therapy (*P* < 0.001). According to the tissue biopsy results, three cases concurrently carried EGFR L858R mutations and one case carried a KRAS mutation in the corresponding tissue samples. Among the T790M mutant-positive patients, 64% (18 of 28) were male and 75% (21 of 28) were diagnosed with adenocarcinoma. The median age was 61 (58–68) years. The numbers of cases with EGFR L858R, 19del, ROS, KRAS, and TP53 mutations were 5, 2, 2, 2, and 1, respectively. There were another two cases with tissue biopsy test results showing no mutations.

### Comparisons of liquid biopsy and tissue biopsy

Furthermore, to evaluate the consistency of the serum cfDNA gene status with tissue molecular test results, the data of the 196 cases that underwent tissue biopsy were investigated. Through tissue biopsy, 58% (114 of 196) of the patients were found to carry EGFR mutations, including thirty-two 19del cases, one T790M case, fifty-six L858R cases, four 19del-L858R co-occurrence cases, three 19del-T790M co-occurrence cases, nine T790M-L858R co-occurrence cases, and nine others. The positive rate of detection by tissue biopsy was much higher than that of liquid biopsy (19.9% vs. 7.6%, respectively, *P* < 0.001) for 19del. However, for T790M, it was slightly lower than that of liquid biopsy (6.6% vs. 7.1%, respectively, *P* = 1.00). The comparison of serum-based 19del analysis using dPCR and tissue testing revealed an overall agreement of 83.67% (164 of 196 cases), kappa = 0.334. The positive predictive value (PPV) was 73.33%. The positive agreement was 28.21% and the negative agreement was 97.45% (Table 2). More specifically, 11 cases were positive for the 19del mutation both in serum and tumor samples, while four cases were detected as mutant only in serum samples. All of the serum-positive-only cases were diagnosed with adenocarcinoma. Two of the patients underwent and benefited from EGFR-TKI therapy, as their previous genetic tests were positive for L858R. The comparison of serum-based T790M analysis using dPCR and tissue testing revealed an overall agreement of 90.31% (177 of 196 cases), kappa = 0.244. The PPV was 28.57%. The positive agreement was 30.77% and the negative agreement was 94.54% (Table 3). More specifically, four cases were positive for the T790M mutation both in serum and tumor samples, while 10 cases were detected as mutant only in serum samples. Three of these serum-positive-only cases underwent EGFR-TKI therapy for more than 20 months, with all of these patients showing disease progression before the drug was changed to osimertinib.

**TABLE 2:** Comparing EGFR 19del status detection using the dPCR assay in serum cfDNA with the tissue molecular test results included in patient medical records (n = 196).

Sample	Serum/dPCR		
	Negative	Positive	Total
Tissue/clinical			
Negative	153	4	157
Positive	28	11	39
Total	181	15	196

EGFR, epidermal growth factor receptor; dPCR, digital polymerase chain reaction; cfDNA, cell-free DNA.

**TABLE 3:** Comparing EGFR T790M status detection using the dPCR assay in serum cfDNA with the tissue molecular test results included in patient medical records (n = 196).

Sample	Serum/dPCR		
	Negative	Positive	Total
Tissue/clinical			
Negative	173	10	183
Positive	9	4	13
Total	182	14	196

EGFR, epidermal growth factor receptor; dPCR, digital polymerase chain reaction; cfDNA, cell-free DNA.

### The correlations between serum EGFR 19del and T790M mutations by dPCR and serum tumor marker levels by ECLIAs

346 results of serum CY211, NSE, ProGRP, and CEA levels, 338 results of SCC levels, 309 results of CA199 and CA125 levels, 306 results of CA153 levels, 304 results of CA724 levels, and 298 results of AFP levels were finally obtained. The median CA199 concentration was approximately two-fold higher in the 19del-positive serum samples compared with the WT serum samples (21.2 vs. 11.0, respectively, *P* = 0.005). The median ProGRP concentration was significantly higher in the T790M-positive serum samples compared with the WT serum samples (51 vs. 46, respectively, *P* = 0.028) (Table 4).

**TABLE 4:** Tumor marker levels of EGFR mutant-positive and EGFR mutant-negative samples.

Tests	dPCR- 19del		*p* value	dPCR-T790M		*p* value
	Negative	Positive		Negative	Positive	
CY211 (ng/mL) (n =346)	2.3 (1.6–4.3)	3.4 (2.1–5.3)	0.147	2.3 (1.6–4.6)	2.6 (2.0–3.7)	0.66
NSE (ng/mL) (n =346)	14.1 (11.9–17.0)	13 (10.7–20.3)	0.91	14.2 (12.1–17.1)	13.4 (11.3–15.6)	0.165
ProGRP (pg/mL) (n =346)	46.7 (35.7–59.2)	43.9 (36.9–58.4)	0.85	46 (35.7–58.5)	51 (45.2–71)	**0.028**
CEA (ng/mL) (n =346)	3.2 (1.8–9.96)	9.8 (2.09–140)	0.084	3.2 (1.8–10.0)	4.1 (2.9–18.9)	0.098
SCCA (ng/mL) (n =338)	1.2 (0.8–1.9)	1.15 (0.6–1.4)	0.115	1.2 (0.8–1.9)	1.2 (0.9–1.8)	0.91
CA199 (U/mL) (n =309)	11 (7.05–20.3)	21.2 (12.0–102.2)	**0.005**	11.1 (7.1–21)	14.9 (7.7–25.6)	0.41
CA125 (U/mL) (n =309)	15.7 (10.3–31.6)	21.7 (14.0–23.4)	0.059	15.8 (10.3–34)	16.5 (12.1–29)	0.70
CA153 (U/mL) (n =306)	15.1 (9.2–23.9)	15.9 (9.1–23.4)	0.77	15.1 (9.2–23)	19.2 (9.4–26.2)	0.45
CA724 (U/mL) (n =304)	2.8 (1.5–7.0)	2.7 (1.5–3.9)	1.00	2.8 (1.5–7.3)	2.6 (2–3.7)	0.50
AFP (ng/mL) (n =298)	3.2 (2.2–4.2)	2.5 (1.8–3.9)	0.164	3.2 (2.2–4.2)	2.9 (2.3–4.2)	1.00

EGFR, epidermal growth factor receptor; dPCR, digital polymerase chain reaction; Cy211, cytokeratin 19 fragment; NSE, neuron specific enolase; ProGRP, progastrin-releasing peptide; CEA, carcinoembryonic antigen; SCCA, squamous cell carcinoma antigen; CA199, carbohydrate antigen 199; CA125, carbohydrate antigen 125; CA153, carbohydrate antigen 153; CA724, carbohydrate antigen 724; AFP, alpha1-fetoprotein. Bold values indicate statistical significance (*p* < 0.05).

**FIGURE 4:**
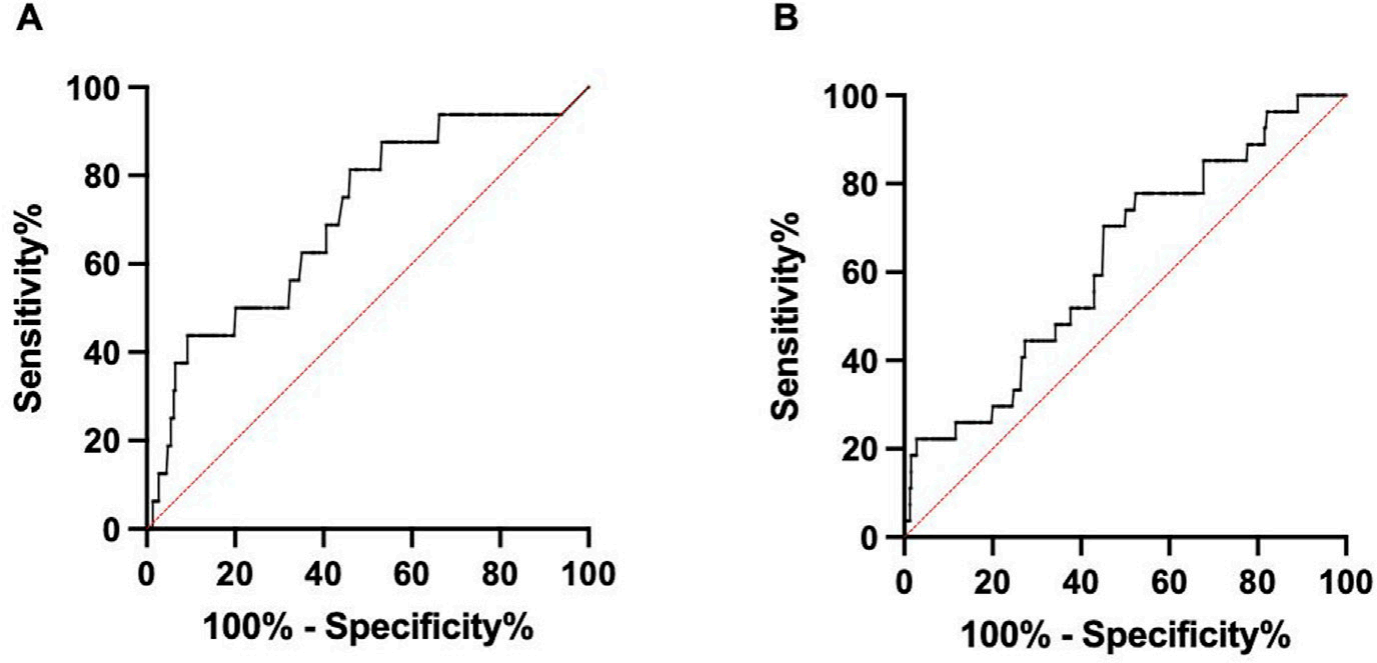
Receiver operating characteristic (ROC) curve analysis of serum tumor marker concentrations in EGFR mutation detection. **(A)** ROC curve of CA199 levels in 19del detection (AUC = 0.707, 95% CI: 0.573–0.841, *P* = 0.005). **(B)** ROC curve of ProGRP levels in T790M detection (AUC = 0.628, 95% CI: 0.521–0.735, *P* = 0.028).

ROC curve analysis was performed to obtain cutoff values of serum tumor marker concentrations that can predict EGFR mutation in serum liquid biopsy. For 19del, the CA199 cutoff value was 11.75 U/mL to reach the dPCR analytical sensitivity threshold of 0.05% mutant (*P* = 0.006), with an area under the ROC curve (AUC) value of 0.707 (95% confidence interval (CI): 0.573–0.841, *P* = 0.005) and a mutant positive rate of 8.78%. For T790M, the ProGRP cutoff value was 45.15 pg/mL to reach the dPCR analytical sensitivity of 0.10% mutant (*P* = 0.012), with an AUC value of 0.628 (95% CI: 0.521–0.735, *P* = 0.028) and a mutant positive rate of 11.11% (Figure 4).

**FIGURE 5:**
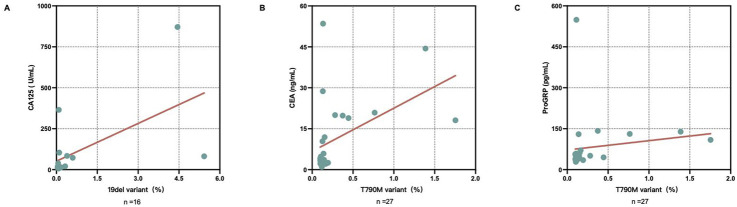
Correlations between the variant rates of epidermal growth factor receptor (EGFR)-mutant samples and serum tumor marker concentrations. **(A)** Positive correlation between the serum carbohydrate antigen 125 (CA125) levels and EGFR 19del variant rate (r = 0.624, *P* = 0.010). **(B)** Positive correlation between the serum carcinoembryonic antigen (CEA) levels and EGFR T790M variant rate (r = 0.531, *P* = 0.004). **(C)** Positive correlation between the serum progastrin-releasing peptide (ProGRP) levels and EGFR T790M variant rate (r = 0.395, *P* = 0.041).

Furthermore, a significant positive correlation was observed between the variant rate and CA125 concentration in 19del mutant-positive serum samples (r = 0.624, *P* = 0.010), while significant positive correlations were found between the variant rate and the CEA and ProGRP concentrations in T790M mutant-positive serum samples (r = 0.531, *P* = 0.004; r = 0.395, *P* = 0.041, respectively) (Figure 5).

### The performance of serum genetic test results by dPCR for identifying disease progression

Table 5 shows the serum test results of EGFR 19del and T7910M mutations in patients with progressive disease and those with stable disease after EGFR-TKI treatment. The EGFR T790M variant rates and copies of patients with progressive disease were significantly higher than those of patients with stable disease (*P* = 0.031, *P* = 0.024, respectively). ROC curve analysis showed that the AUC values were 0.6091 (95% CI: 0.5091–0.7091, *P* = 0.036 for the variant rate and 0.6135 (95% CI: 0.5129–0.7140, *P* = 0.030) for the variant copies. The Youden index values were 0.25 and 0.239, respectively. Additionally, the optimal cutoff values for the serum T790M variant rate and copies determined by ROC analysis were 0.0248% (with 64.2% sensitivity and 60.8% specificity) and 0.0247 copies/μL (with 60.4% sensitivity and 63.5% specificity).

**TABLE 5:** The dPCR results of patients with progressive disease and stable disease after EGFR-TKI treatment.

EGFR mutations	PD (n = 53)	SD (n = 74)	*p* value
19del			
Variant rate (%)	0 (0–0.0272)	0 (0–0)	0.31
Variant (copies/μL)	0 (0–0.0266)	0 (0–0)	0.50
Wild type (copies/μL)	92.8651 (51.7457–179.8197)	84.1420 (44.9914–147.6963)	0.53
T790M			
Variant rate (%)	0.0446 (0–0.0683)	0.0147 (0–0.0555)	**0.031**
Variant (copies/μL)	0.0471 (0–0.0740)	0.0238 (0–0.0546)	**0.024**
Wild type (copies/μL)	72.8253 (43.4522–147.4915)	67.7830 (35.0735–121.2496)	0.41

dPCR, digital polymerase chain reaction; PD, progressive disease; SD, stable disease; EGFR-TKI, epidermal growth factor receptor tyrosine kinase inhibitor. Bold values indicate statistical significance (*p* < 0.05).

## Discussion

Compared with patients with WT EGFR, patients harboring EGFR TK domain mutations benefit more from EGFR-TKI therapy [2]. Accurate detection of EGFR mutations in NSCLC patients is therefore crucial for therapeutic stratification and longitudinal treatment monitoring. While tissue biopsy remains the diagnostic gold standard, its clinical utility is constrained by inherent limitations. In addition to its invasive nature, it provides only a static and spatially-limited assessment of the disease at the moment of the surgical procedure. Indeed, studies have shown that 27%–31% of NSCLC patients are unable to provide a biopsy sample suitable for EGFR mutation analysis at diagnosis or following disease progression [11]. This statistic increased to 70% for patients with locally advanced or metastatic disease at the time of NSCLC diagnosis [2]. Furthermore, a subset of patients with progressive disease decline repeat biopsies for molecular profiling despite clinical recommendations. In contrast, liquid biopsy offers a minimally invasive alternative enabling rapid, cost-effective, and real-time cancer longitudinal monitoring method that can capture tumor heterogeneity. Nevertheless, technical challenges persist in circulating cfDNA analysis due to its low concentration, genomic DNA contamination, and high fragmentation [13]. Additionally, inter-platform variability in dPCR methodologies - particularly regarding reference interval thresholds and gene panel configurations - complicates result standardization across laboratories.

The data in this study described the performance characteristics of a novel dPCR assay platform for detecting EGFR 19del and T790M mutations in serum-derived cfDNA from NSCLC patients. This work demonstrated that the dPCR assay could achieve absolute quantification of mutant alleles according to Poisson distribution. The variant copy concentrations showed statistically significantly differences between EGFR mutant and WT samples. As was well known dPCR adopted the concept of “divide and conquer”, which endowed it with excellent anti-interference ability. Based on the data provided, the dPCR method was found to be capable of detecting EGFR gene mutations in serum cfDNA samples that it could even detect gene mutations missed by tissue biopsy in some cases. However, the comparisons of gene status in serum cfDNA samples by dPCR assay and in tissue samples from patient clinical medical records suggested a rather high negative agreement and rather low positive agreement, which indicated that the assay was useful for ruling out mutations, but its sensitivity for detecting positives may be insufficient. The moderate performance of the current dPCR technology was indeed not as good as previously reported. In particular, further refinement might be necessary to improve the tests’ low PPV in order to meet the clinical needs. It must be admitted that the differences in EGFR mutation detection capabilities between serum and tissue samples did exist that could not be ignored. There were several possible reasons for this. First, for the purpose of measuring multiple other cancer associated protein markers, we chose serum samples instead of the usual plasma samples for cfDNA extraction. The amount of ctDNA in each 1 mL serum sample was limited, with a potentially insufficient amount for dPCR amplification. Previous studies [14–17] have demonstrated that there are very low concentrations of cfDNA (1–5 ng/mL in healthy individuals and 5–1,500 ng/mL in cancer patients) and ctDNA (only a small fraction; <1% of the total cfDNA) in the bloodstream. Furthermore, the proportion of ctDNA present in a blood sample is related to the concordance of mutation profiles between tissue and blood. Insufficient materials could lead to false negative results. Second, the tissue and blood collection processes were not conducted at the same time in our study, and with long intervals. Jeffrey et al. [18] determined that a shorter time interval between tissue and blood collection was associated with increased concordance. As time goes by, secondary mutations and genotypic shifts might occur with recurrent tumors. This temporal genomic heterogeneity can have a substantial impact on the subsequent treatment outcomes [19].

Our study evaluated the potential relationship between serum biomarker levels and mutation detection in serum cfDNA by dPCR. Interestingly, we found that enhanced levels of certain tumor markers, specifically CA199 and ProGRP, could be potentially used as simple and practical clinical predictors of EGFR 19del and T790M mutation detection by dPCR liquid biopsy assay. Individuals with a serum CA199 level higher than 11.75 U/mL or ProGRP level higher than 45.15 pg/mL had an increased likelihood of EGFR mutation. However, the AUC values were below 0.8, which indicated that although the test can differentiate between mutation-positive and -negative cases to some extent, it might miss some positives or generate some false positives. On the other hand, significant positive correlations between serum CA125, CEA, and ProGRP levels and the variant rates in the EGFR mutant samples were observed. ProGRP is a tumor marker often used for the differential diagnosis of SCLC and NSCLC. Increasing ProGRP levels can help rule out NSCLC. Kudo et al. [20] reported that high ProGRP levels is also associated with neuroendocrine differentiation components of NSCLC. Kato et al. [21, 22] reported cases of NSCLC to SCLC transformation following EGFR-TKI treatment in patients whose ProGRP levels increased with disease progression. Our study further expanded the clinical application potential of this tumor marker. Serum tumor markers partly reflect the condition of ctDNA. A high proportion of ctDNA in the blood may enhance the mutation detection capability. Higher serum tumor marker levels were predictive of increased concordance in treatment-related genes between paired tissue and plasma samples [17]. It was suggested that patients with higher serum CEA and CA199 levels had a significantly higher disease control rate and longer survival time with EGFR-TKI treatment [23].

It was suggested that for patients who underwent EGFR-TKI therapy, T790M mutation detection in serum had a predictive value of disease progression, even when the variant rate was lower than the corresponding cutoff value. More specifically, disease progression might present when the serum T790M variant rates or copies were higher than 0.0248% or 0.0247 copies/μL, respectively. The secondary EGFR T790M mutation constitutes the predominant acquired resistance mechanism to first- and second-generation TKIs [24]. The data indicated the clinical significance of mutation-negative results.

Our study indicated that the dPCR technology was indeed helpful in reducing the rate of missed diagnosis and was valuable for improving the level of clinical diagnosis, although the current effect was still not entirely satisfactory. There were certain limitations of this study that should be noted. First, this study was conducted in a single center, which resulted in a limited sample size and a possibility of bias. Thus, the results cannot truly reflect the dPCR assay performance for detecting EGFR 19del and T790M mutations among the population. Second, the low cfDNA concentration and high possibility of genomic DNA contamination in serum samples could result in an insufficient quantity of alleles for PCR amplification. Future studies that include an increased plasma volume and magnetic bead extraction method in place of the column method are needed to improve the cfDNA extraction efficiency. Third, the performance index data of the dPCR method, including sensitivity and specificity values, were difficult to accurately obtain because it was not feasible to perform tissue rebiopsy for further comparison. The moderate performance of the dPCR assay using serum suggested that further refinement or complementary markers might be necessary to improve the tests’ PPV, especially in populations with lower mutation prevalence.

In conclusion, serum-based dPCR liquid biopsy assay demonstrates intermediate diagnostic capability for EGFR mutation monitoring. The assay should be reserved as an ancillary diagnostic tool in clinical scenarios characterized by contraindications to tissue biopsy or insufficient tumor material availability. Although further technical refinement is required to improve positive predictive performance, the assay is with good application value and prospects. Notably, its potential clinical utility lies in indicating possible disease transformation, oncogene drift or secondary mutations during longitudinal monitoring of NSCLC progression. To enhance practical implementation, combined detection of certain serum tumor markers emerges as a pragmatic triage strategy. This may facilitate the identification of subgroups most likely to benefit from EGFR testing, thereby improving the cost-effectiveness of precision oncology workflows through targeted patient stratification.

## Data Availability

The original contributions presented in the study are included in the article/supplementary material, further inquiries can be directed to the corresponding authors.

## References

[1] Qi J Li M Wang L Hu Y Liu W Long Z National and subnational trends in cancer burden in China, 2005-20: an analysis of national mortality surveillance data. The Lancet Public Health (2023) 8:e943–e955. 10.1016/s2468-2667(23)00211-6 38000889

[2] da Cunha Santos G Shepherd FA Tsao MS. EGFR mutations and lung cancer. Annu Rev Pathol (2011) 6:49–69. 10.1146/annurev-pathol-011110-130206 20887192

[3] Franzi S Seresini G Borella P Raviele PR Bonitta G Croci GA Liquid biopsy in non-small cell lung cancer: a meta-analysis of state-of-the-art and future perspectives. Front Genet (2023) 14:1254839. 10.3389/fgene.2023.1254839 38116291 PMC10728669

[4] Gou LY Wu YL. Prevalence of driver mutations in non-small-cell lung cancers in the People’s Republic of China. Lung Cancer (Auckland, N.Z.) (2014) 5:1–9. 10.2147/lctt.s40817 28210137 PMC5217505

[5] Ettinger DS Wood DE Aisner DL Akerley W Bauman JR Bharat A Non-small cell lung cancer, version 3.2022, NCCN clinical practice guidelines in oncology. J Natl Compr Cancer Netw (2022) 20:497–530. 10.6004/jnccn.2022.0025 35545176

[6] Lu Z Yi Y Wang L Luo Y Luo D Xiong L Non-small cell lung cancer cells with uncommon EGFR exon 19delins variants respond poorly to third-generation EGFR inhibitors. Translational Oncol (2024) 39:101834. 10.1016/j.tranon.2023.101834 PMC10728704 38006760

[7] Kim C Liu SV. First-line EGFR TKI therapy in non-small-cell lung cancer: looking back before leaping forward. Ann Oncol (2019) 30:1852–5. 10.1093/annonc/mdz415 31613313

[8] Casagrande GMS Silva Md O Reis RM Leal LF. Liquid biopsy for lung cancer: up-to-date and perspectives for screening programs. Int J Mol Sci (2023) 24:2505. 10.3390/ijms24032505 36768828 PMC9917347

[9] Bao H Min L Bu F Wang S Meng J. Recent advances of liquid biopsy: interdisciplinary strategies toward clinical decision-making. Interdiscip Med (2023) 1:e20230021. 10.1002/inmd.20230021

[10] Zhou Y Shen L. Clinical application and challenges of liquid biopsy biomarkers in non-small cell lung cancer. Lab Med (2023) 38:807–11. 10.3969/j.issn.1673-8640.2023.09.001

[11] Normanno N Denis MG Thress KS Ratcliffe M Reck M. Guide to detecting epidermal growth factor receptor (EGFR) mutations in ctDNA of patients with advanced non-small-cell lung cancer. Oncotarget (2017) 8:12501–16. 10.18632/oncotarget.13915 27980215 PMC5355360

[12] Hao J Liu M Zhou Z Zhao C Dai L Ouyang S. Predicting epidermal growth factor receptor (EGFR) mutation status in non-small cell lung cancer (NSCLC) patients through logistic regression: a model incorporating clinical characteristics, computed tomography (CT) imaging features, and tumor marker levels. PeerJ (2024) 12:e18618. 10.7717/peerj.18618 39650554 PMC11623057

[13] Martignano F. Cell-free DNA: an overview of sample types and isolation procedures. Methods Mol Biol (2019) 1909:13–27. 10.1007/978-1-4939-8973-7_2 30580420

[14] Merker JD Oxnard GR Compton C Diehn M Hurley P Lazar AJ Circulating tumor DNA analysis in patients with cancer: American society of clinical oncology and college of American pathologists joint review. J Clin Oncol (2018) 36:1631–41. 10.1200/jco.2017.76.8671 29504847

[15] Diaz LA Jr Bardelli A. Liquid biopsies: genotyping circulating tumor DNA. J Clin Oncol. (2014) 32(6):579–586. 10.1200/JCO.2012.45.2011 24449238 PMC4820760

[16] Ranucci R. Cell-free DNA: applications in different diseases. Methods Mol Biol (2019) 1909:3–12. 10.1007/978-1-4939-8973-7_1 30580419

[17] Jiao XD Ding LR Zhang CT Qin BD Liu K Jiang LP Serum tumor markers for the prediction of concordance between genomic profiles from liquid and tissue biopsy in patients with advanced lung adenocarcinoma. Transl Lung Cancer Res (2021) 10:3236–50. 10.21037/tlcr-21-543 34430361 PMC8350084

[18] Thompson JC Yee SS Troxel AB Savitch SL Fan R Balli D Detection of therapeutically targetable driver and resistance mutations in lung cancer patients by next-generation sequencing of cell-free circulating tumor DNA. Clin Cancer Res (2016) 22:5772–82. 10.1158/1078-0432.ccr-16-1231 27601595 PMC5448134

[19] Fang Q Wan X D'Aiello A Sun H Gu W Li Y Temporal genomic heterogeneity guiding individualized therapy in recurrent non-small cell lung cancer. Front Oncol (2023) 13:1116809. 10.3389/fonc.2023.1116809 37503313 PMC10368968

[20] Kudo K Ohyanagi F Horiike A Miyauchi E Yanagitani N Hoshi R Clinicopathological findings of non-small-cell lung cancer with high serum progastrin-releasing peptide concentrations. Lung Cancer (2011) 74:401–4. 10.1016/j.lungcan.2011.03.019 21529988

[21] Kato Y Tanaka Y Hino M Gemma A. ProGRP as early predictive marker of non-small-cell lung cancer to small-cell lung cancer transformation after EGFR-TKI treatment. Respir Med Case Rep (2019) 27:100837. 10.1016/j.rmcr.2019.100837 31016132 PMC6468187

[22] Watanabe S Sone T Matsui T Yamamura K Tani M Okazaki A Transformation to small-cell lung cancer following treatment with EGFR tyrosine kinase inhibitors in a patient with lung adenocarcinoma. Lung Cancer (2013) 82:370–2. 10.1016/j.lungcan.2013.06.003 24012411

[23] Pan JB Hou YH Zhang GJ. Correlation between efficacy of the EGFR tyrosine kinase inhibitor and serum tumor markers in lung adenocarcinoma patients. Clin Lab (2014) 60:1439–47. 10.7754/clin.lab.2013.131002 25291939

[24] Ko B Paucar D Halmos B. EGFR T790M: revealing the secrets of a gatekeeper. Lung Cancer Targets Ther (2017) 8:147–59. 10.2147/lctt.s117944 PMC5640399 29070957

